# Multiple suicide attempts associated with addiction to tramadol

**DOI:** 10.1186/s12991-022-00401-6

**Published:** 2022-07-01

**Authors:** Bénédicte Nobile, Marine Bonnin, Emilie Olié, Philippe Courtet

**Affiliations:** 1 Department of Emergency Psychiatry and Acute Care, Lapeyronie Hospital CHU Montpellier, Montpellier, France; 2 IGF, Univ. Montpellier, CNRS, INSERM, Montpellier, France; 3 FondaMental Foundation, Montpellier, France

## Abstract

**Background:**

The opioid tramadol is used as analgesic drug, and more recently was also proposed for the management of major depressive disorder. However, growing evidence suggests a link between opioid system dysfunction and suicidal behaviors, raising the question of tramadol use in view of the high addictive and suicidal risk. Here, we present the case of a young adult woman with multiple suicide attempts related to tramadol addiction.

**Case presentation:**

A 25-year-old woman was admitted for suicide attempt by phlebotomy in the Department of Psychiatric Emergency and Acute Care, Montpellier (France), in March 2020. The suicide attempt occurred 3 days after an abrupt tramadol withdrawal. In 2018, due to spinal disc herniation, she had a first prescription of tramadol to which she became addicted. The patient described an effect on psychological pain and suicidal ideation. However, she had to increase tramadol dose to obtain the desired effects, and for several months her intake was 2 000 mg per day. When she could not obtain tramadol any longer, suicidal ideation and psychological pain increased, leading to the suicide attempt. At the time of a worldwide opioid crisis that contributes to increasing suicidal behaviors, this case raises questions about tramadol prescription (often considered to be less addictive and with lower abuse potential) to individuals at risk of suicide.

## Introduction

In the last 20 years, the United States of America (USA) have been facing an opioid crisis that has led to an increase in mortality by overdose and suicide [1]. This crisis is now spreading in Europe [2], as indicated by the rise of opioid prescriptions between 2004 and 2016, especially in Northern and Western European countries [3]. In France, hospitalizations due to opioid overdose increased by 128% and death due to opioid overdose by 161% between 2000 and 2015 [4].

According to data from the USA Centers for Disease Control and Prevention, deaths due to drug overdose and suicide increased from 41,364 in 2000 to 110,749 in 2017, and opioids were implicated in one third of suicides by overdose [5]. This suicide risk increase is not only due to the access to opioids as a lethal mean. ﻿Indeed, it has been shown that the suicide risk, whatever the method, is higher among individuals receiving higher opioid doses [6]. Consequently, the Department of Veterans Affairs and Clinical Practice Guidelines for the Management of Opioid Therapy defined high suicidal risk as a contraindication for opioid therapy. Prescription of opioid analgesics (but not of non-opioid analgesics) has been associated with lifetime history of suicide attempt (SA) in cohorts of elderly people from the general population, independently of the physical pain level and health status [7, 8]. Moreover, growing evidence suggests a link between opioid system dysfunction and suicidal behaviors [9].

Besides its analgesic use, recent studies investigated tramadol antidepressant properties for the management of major depressive disorder (MDD) [10–12]. Tramadol is a mu-opioid receptor agonist with serotoninergic and noradrenergic activities through norepinephrine and serotonin reuptake inhibition (like done by some antidepressants). Tramadol is often considered to be less addictive and with lower abuse potential than other opioids [13], but this remains debated [14, 15]. Indeed, in Finland, tramadol was implicated in 29.4% of all opioid misuse cases between 2010 and 2011, more than codeine (16.3%), fentanyl (14.5%) and oxycodone (6.9%) [16]. In Ireland, deaths linked to tramadol use increased from 9 to 40% of all deaths linked to drug misuse between 2001 and 2011 [17]. Among adolescents and young adults (10–29 years of age) in Ohio (USA), tramadol was implicated in 24.8% of all intentional opioid poisoning cases between 2002 and 2014 [18].

These findings raise the question of tramadol use as antidepressant and also more generally about its use in view of the high addiction and suicide risks. Here, we present the case of a young adult woman with multiple SA related to tramadol addiction. The purpose of this case report is to alert practitioners about the risk of prescription of Tramadol particularly in patients at risk of suicide. Indeed, Tramadol prescription has increased of 68% between 2006 and 2017 and is now the first opioid prescribed in France but also in some others European countries, such as Germany, Italia, Spain and Danemark [19]. Thus, evaluating potential risk of addiction especially within suicidal patients may be primordial to prevent addiction and worsening of suicidal risk, as we will see in this case report.

## Case presentation

A 25-year-old woman was admitted following a SA by phlebotomy to the Department of Psychiatric Emergency and Acute Care, Montpellier (France), in March 2020. She was single, without children, living with her parents, and with good social support. She did not report history of childhood trauma. She never consulted a psychiatrist or a general practitioner for mental disorders and was never hospitalized in a psychiatric unit despite three previous SA: two aborted SA by hanging (in 2012 in a context of grief, and in 2014 in a context of romantic difficulties) and one SA by phlebotomy in 2019 after being informed about her sister’s miscarriage.

Moreover, she reported spinal disc herniation in 2018 and three tonic–clonic seizures in 2019 and 2020 (unknown context) leading to a prescription of lamotrigine (100 mg per day) by a neurologist that she never took. For the pain caused by the herniated disc, after the failure of different analgesics (the patient was unable to remember their names), she was prescribed tramadol sustained release tablets (100 mg) to take when needed (maximum of 2 tablets per day). She developed an addiction to tramadol. She also reported addiction to tobacco, but not to alcohol or other substances.

## Investigations

At admission, the patient was assessed by a psychiatrist who diagnosed MDD using the Mini International Neuropsychiatric Interview (MINI-5). The depressive symptomatology was moderate: the scores of the 30-item Inventory of Depressive Symptomatology Clinician-rated and Montgomery–Åsberg Depression Rating Scale were 27/84 and 24/60, respectively. The suicidal ideation (SI) intensity was severe (Columbia-Suicide Severity Rating Scale score for SI = 5/5). The current SA was triggered by tramadol withdrawal for 3 days. The day following the drug discontinuation, she experienced SI resurgence concomitantly with tramadol craving. She called the emergency department, but she did not receive any support. Two days later, she went to a pharmacy with a falsified prescription that was recognized by the pharmacist who informed her about the judiciary consequences of her action. She went home, where she attempted suicide by phlebotomy.

An addictologist and a pharmacist evaluated her tramadol addiction. The patient started to use tramadol as analgesic in 2018 due to her herniated disc. At the beginning, the physical pain was relieved. She then started to take tramadol tablets more frequently to prevent pain. She also reported that tramadol decreased her psychological pain. She felt more energic, less anxious and depressed, relieved of negative thoughts and SI. She saw the “life in pink” and had more ideas about new projects. This was not linked to a hypomanic state, because this patient had no diagnosis of bipolar disorder. However, the patient progressively increased the daily dose of tramadol to obtain the same psychological effect: first, five slow-release tablets per day (500 mg daily) until the end of 2018, then up to 1000 mg per day (the maximum dose is 450 mg per day) using tramadol oral solution in 2019, and finally 2000 mg per day at the end of 2019 and until her SA, triggered by tramadol withdrawal. At the beginning of the hospitalization, she presented withdrawal symptoms (e.g., impatience, anxiety, diarrhea, and chills) that disappeared rapidly with diazepam and the initiation of an opioid substitution treatment. All laboratory parameters were normal.

## Differential diagnosis

Except the current MDD and tramadol addiction, no other psychiatric disorder or somatic disease was found.

## Treatment

For the MDD, paroxetine (20 mg per day; a serotonin reuptake inhibitor) with diazepam (5 mg; benzodiazepine), if needed for anxiety and/or insomnia (maximum two tablets per day for 15 days), were prescribed. The opioid substitution treatment with methadone (20 mg per day for the first 3 days and then 5 mg each 3 days) was initiated during the hospitalization. Although the patient did not have tonic–clonic seizures, lamotrigine at low dose with a stepwise increase was reintroduced by the psychiatrists. After 10 days, she was transferred to another unit to continue the care initiated in the Psychiatric Emergency and Acute Care department.

## Outcome and follow-up

Few days after her hospital discharge, the patient was admitted to a private psychiatric clinic. However, due to the COVID-19 pandemic, she was discharged earlier than planned, after 10 days of hospitalization. Fifteen days later, she attempted suicide by voluntary drug poisoning with diazepam in the context of a conflict with her parents. She was readmitted to the Emergency Psychiatry and Acute Care department in May 2020. She was not taking tramadol at that time. Considering this new SA shortly after discharge from hospital, an interview with a practitioner specialized in borderline personality disorder was carried on and this new diagnosed has been done, thus quetiapine (150 mg per day) was introduced in addition to the other drugs. She was then hospitalized in a private psychiatric clinic for 3 months (until July 2020). In September 2020, the patient stopped all psychotropic treatments and follow-up. At a party, she took tramadol and in the following days, she progressively increased the intake through black market purchases. For 3 months, she took 500 mg tramadol per day at wake-up time, but with only a partial effect on SI. At the end, tramadol did not have any effect on psychological pain and SI. The patient reported ruminations about her grandfather’s death (often close to Christmas time). At the end of November 2020, she attempted suicide again by voluntary drug poisoning with paroxetine. She was readmitted to the Emergency Psychiatry and Acute Care department, where all psychotropic drugs and methadone were re-introduced. Since this last hospitalization, she never came to the follow-up visits that were organized and never answered to the psychiatrist’s telephone calls. She did a new SA by phlebotomy in March 2021, but we do not have any information about the context.

## Conclusions and discussion

This case report indicates that tramadol prescription to suicidal patients can be problematic and can lead to dramatic consequences. Tramadol was first prescribed to relieve physical pain but was misused by the patient to reduce her psychological pain. When she could no longer obtain this drug and the effect on psychological pain, the patient did a SA. Thus, clinicians should be careful when prescribing tramadol to patients at risk for SI/SA. Indeed, growing evidence suggest a dysfunction of the opioid system in patients with suicidal behavior [9]. For instance, post-mortem studies on brain samples from patients who committed suicide found an increased mu-opioid receptor (MOR) density in the frontal cortex and caudate compared with controls (death by other causes) [20–22]. Furthermore, the single nucleotide polymorphism A118G in the *MOR* gene is associated with higher sensitivity to social rejection (an important risk factor of suicidal behavior [23]) and treatment-emergent SI [24]. Thus, prescribing opioids to patients with suicidal vulnerability could contribute to suicidal behavior emergence.

In conclusion, the case of this patient could serve as an example to highlight the need of caution when prescribing opioids as analgesics, even tramadol that is considered to have a lower addiction potential than other opioids. Finally, although tramadol has been proposed as an antidepressant drug, its use for this indication should be more thoroughly studied and may need closer supervision.

## Data Availability

Not applicable.
